# Palliative Surgery for Rare Cases of Anterior Urethral Metastasis in Prostate Cancer

**DOI:** 10.1155/2014/584957

**Published:** 2014-08-05

**Authors:** Enrique Gómez Gómez, Jose Carlos Carrasco Aznar, Maria del Mar Moreno Rodríguez, José Valero Rosa, Maria José Requena Tapia

**Affiliations:** ^1^Department of Urology, Reina Sofia University Hospital, Avenida Menendez Pidal s/n, 14004 Cordoba, Spain; ^2^Department of Pathology, Reina Sofia University Hospital, Avenida Ramirez de Arellano No. 1, 14002 Cordoba, Spain

## Abstract

Penis metastasis from prostate cancer is very rare, and its management varies from case to case as there are very few cases reported in the literature. We describe a patient with prostate cancer treated with radiotherapy and androgen deprivation therapy who presented with urethral bleeding as a symptom of anterior urethral metastasis during followup. We propose a way to manage this and review the literature.

## 1. Introduction

Metastatic prostate cancer in the anterior urethra is very rare. The main locations of metastasis are bone, distal lymph, liver, and lung [1]. When the penis is affected, metastasis in the corpus cavernous and foreskin is more frequent than in the urethra or corpus spongiosum. 28% of penile metastasis is due to prostate cancer [2–4].

The standardization of its treatment is very difficult due to the low incidence of this disease and its poor prognosis. However, its bothersome symptoms warrant a solution.

## 2. Case Report

A 70-year-old Caucasian male with no family history of prostate cancer presented with initial diagnosis of localized high risk prostate cancer in October 2008. The PSA value when he was diagnosed was 47 ng/mL and he underwent a nonpathologic digital rectal examination (DRE). A biopsy was carried out with a histologic diagnosis of Gleason 7 (3 + 4) bilateral, affecting a maximum of 20% of the sample in the left side. To decide the most appropriate treatment an extension study (computerized tomography and bone scintigraphy) was performed which resulted negative. For this reason, we began a combined treatment of radiotherapy and androgen deprivation therapy (nadir PSA: 0.01 ng/mL).

**Figure 1:**
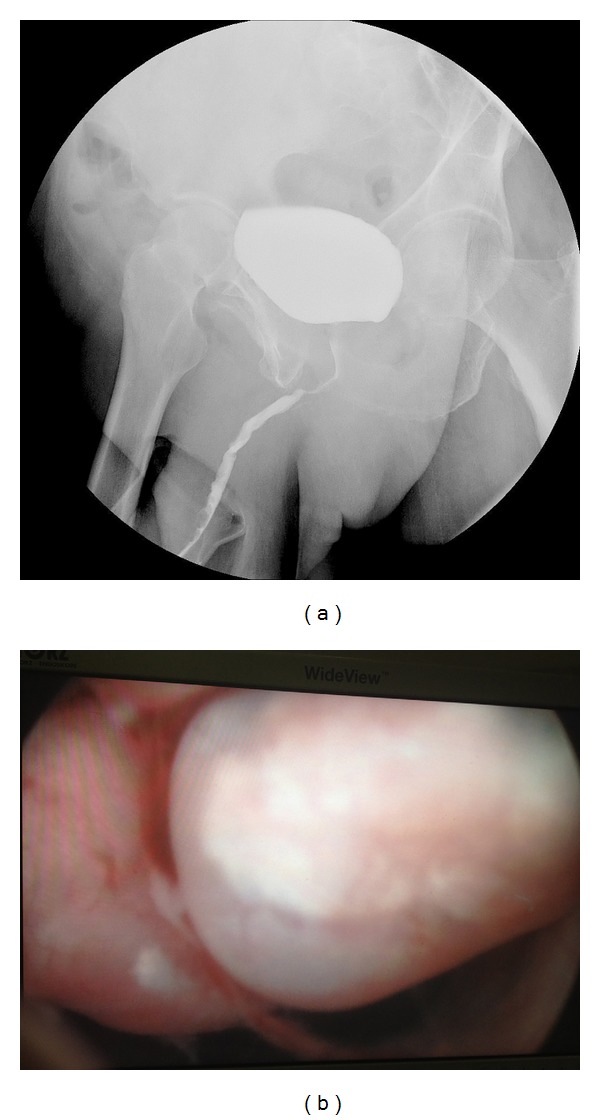
Diagnostic images. (a) Urethral stenosis. Voiding urethrography. (b) Nodular tumour appearance in penile urethra.

**Figure 2:**
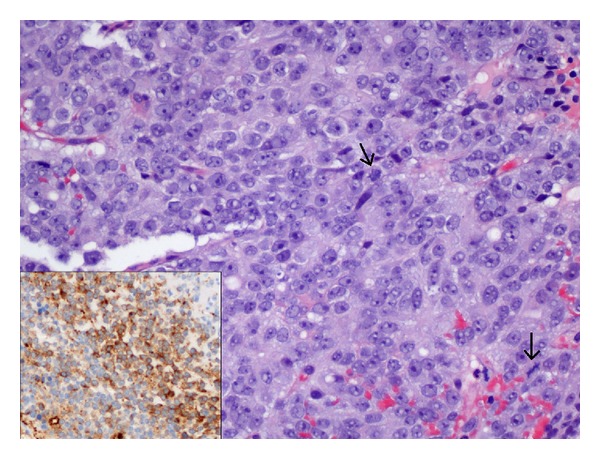
Metastasis in penile urethra: tumour composed of solid sheets or single cells with prominent nucleoli and several mitotic figures (arrows) (hematoxylin and eosin, 200x). Prostate specific antigen in the cytoplasm (inset) (100x): Gleason 5 + 5.

After three years of androgen deprivation therapy with Goserelin (January 2012), the PSA value was 1.7 ng/mL, despite testosterone levels being in castration range. At this time, the patient reported bothersome LUTS and urethral bleeding so a cystoscopy and retrograde cystourethrogram were performed, showing a nodular lesion between the distal bulbar urethra and proximal penile urethra (Figure 1). The physical exploration did not suggest infiltration of corpus spongiosum. The biopsy showed prostate adenocarcinoma Gleason 10 (5 + 5) (Figure 2).

The PSA rose to 2.37 ng/mL, so bicalutamide 50 mg was added to the treatment. Further bone scintigraphy and computerized tomography were negative and the PSA dropped to 1.19 ng/mL 4 months later.

**Figure 3:**
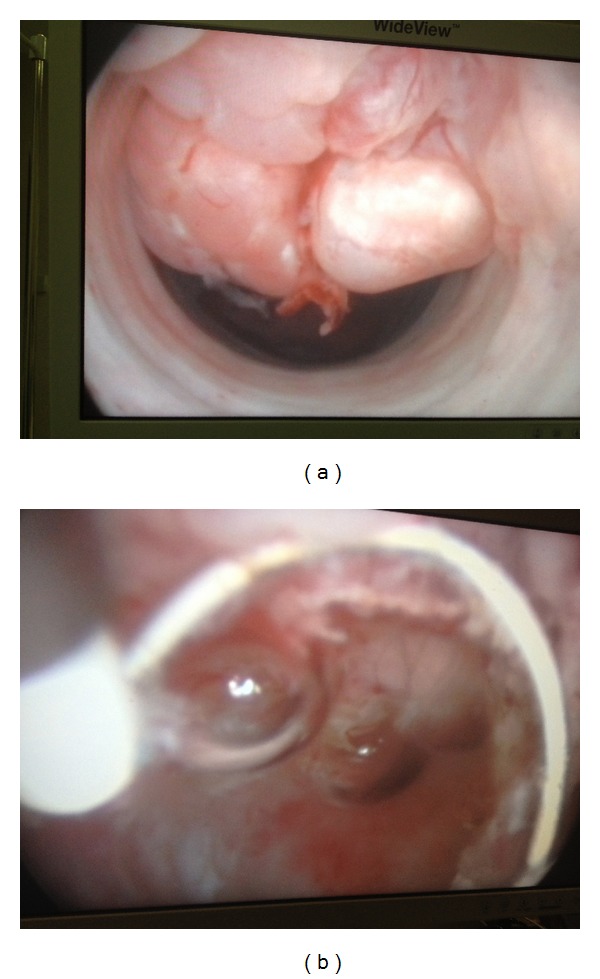
First step: endoscopic surgery. (a) Appearance of sessile-nodular lesion between the distal bulbar urethra and proximal penile urethra. (b) Surgical bed after endoscopic resection.

A decision was taken to carry out an endoscopic resection (June 2012) of the lesions in order to avoid the bothersome symptoms of urethral bleeding and LUTS (Figure 3). The initial outcome was favorable but the symptoms returned within three months.

Given the negative outcome of the surgery, the patient asked for a more definitive solution and so we continued with further palliative surgery after confirming the recurrence of the nodular lesions with a cystoscopy. No other additional work-up was performed before the final surgery.

**Figure 4:**
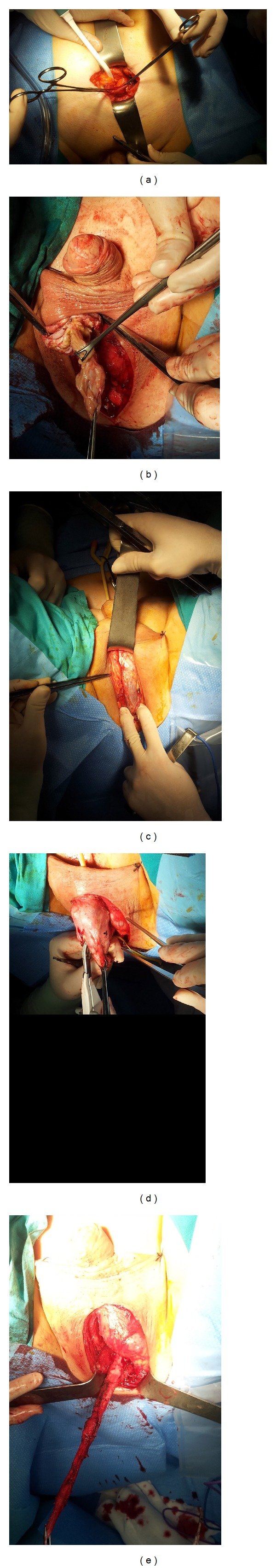
Final surgical step. From upper-left to lower-right: (a) performing vesicostomy; (b) subalbuginea orchiectomy; ((c), (d), and (e)) sequential steps of urethrectomy.

**Figure 5:**
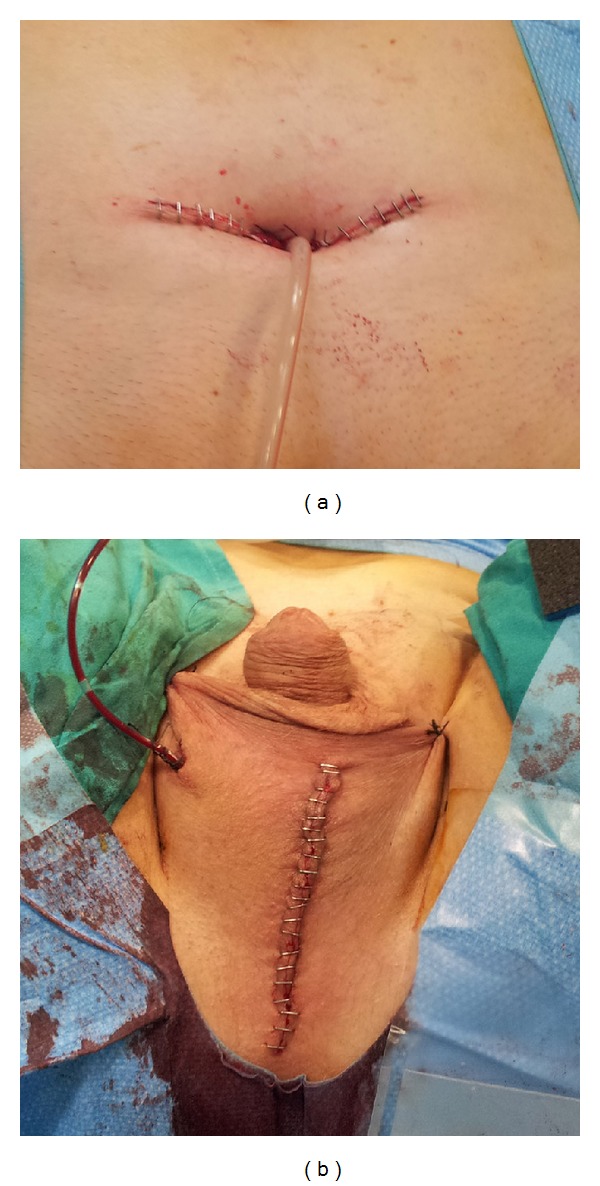
Postoperative appearance. (a) Final appearance of vesicostomy. (b) Final appearance of incision for subalbuginea orchiectomy and urethrectomy.

An anterior urethrectomy, subalbuginea orchiectomy, and a vesicostomy (January 2013) (Figures 4 and 5) according to the technique described by Blocksom were performed. The subalbuginea orchiectomy was performed in order to avoid the need for further injections of GnRH-analogues.

For the urethrectomy, an exaggerated lithotomy position provided optimal exposure for total urethrotomy and a midline perineal incision was made. The corpus spongiosum was mobilized circumferentially at the level of the bulbous urethra. The penis was essentially invaginated into penile skin and the dissection was completed to the base of the glans. The penis returned to its normal position and the distal urethra was excised in order to remove the entire urethra en bloc. For the proximal excision, the urethra was detached from the corpus cavernosum and dissection was completed up to the urogenital diaphragm. The proximal urethral stump was oversewn.

A histopathological examination showed the following.

- The urethra: metastatic prostate adenocarcinoma Gleason (5 + 5) widely infiltrating the lamina propria and focally the corpus spongiosum. It presented signs of lymphovascular invasion.
- The testes: both showed testicular atrophy.

The disease evolution was negative with metastatic pelvic lymphadenopathies. They were visible in a further computerized tomography carried out two months after the surgery because of an increase in the PSA.

The symptoms disappeared after surgery and 6 months later the patient has had no recurrence of these symptoms.

Despite the good functional status of the vesicostomy, the patient preferred to continue using the catheter.

## 3. Discussion

Metastatic routes of prostate cancer to the corpus cavernous or the anterior urethra have been postulated, including direct extension through the urine, implantation by instrumentation, lymphatic spread, and dissemination through the blood stream [4–6].

In our case, it is difficult to determine the exact method of dissemination, although it is probably through the blood stream due to the radiotherapy treatment.

There have been less than 15 published cases, most of them with previous instrumentation [2, 7, 8]. The clinical manifestation varied from a palpable nodule to LUTS or urethral bleeding. Some cases reported a previously diagnosed metastatic prostate cancer or castration resistant prostate cancer (CRPC) [7]. Our patient's PSA levels were controlled but bothersome urethral bleeding with LUTS began.

The diagnosis depends on the clinical manifestation, symptoms such as LUTS, hematuria, and/or urethral bleeding, and the patient reaching a CRPC state. Diagnostic tests such as cystography, cystoscopy, laboratory analysis, and magnetic resonance are carried out to determine the diagnosis, but the most relevant study is of a histological sample [9–11]. In our case, the physical examination, a cystoscopy, and a histological study were enough to secure the correct diagnosis.

The different options for treatment depend on the degree of the metastasis and the symptoms. It should be taken into account that, at this stage of the disease, a radical or curative treatment is not possible, and androgen deprivation therapy or chemotherapy and new hormonal drugs are the basis of the treatment [2, 10]. For local metastasis disease the possibilities varied among the authors: local brachytherapy [12], local endoscopic resection of the urethral tumour [13], urethrectomy, or penectomy [11].

In our case, we decided to carry out a step therapy starting with the less invasive surgery (endoscopic resection), but because a definitive solution was not reached, an anterior urethrectomy was performed. We also carried out a subalbuginea orchiectomy in order to be able to discontinue medical treatment and to ensure the urine derivation, a vesicostomy according to the technique described by Blocksom [14].

As far as we know this is the first case published in the literature managed with this step therapy and this type of definitive surgery. We consider this to be an effective way of managing those patients whose prognosis in the medium-term is negative [2].
